# Clinical relevance, classification, and risk factors for stress shielding in total shoulder arthroplasty: a systematic review and meta-analysis of clinical outcomes

**DOI:** 10.1530/EOR-2025-0139

**Published:** 2026-02-04

**Authors:** Manuel Kramer, Bernhard Jost, Moritz Lebe, Davide Previtali

**Affiliations:** ^1^ Department of Orthopaedics and Traumatology, HOCH Health Ostschweiz, Kantonsspital St. Gallen, St. Gallen, Switzerland; ^2^ Department of Orthopaedics and Traumatology, Ospedaliero Cantonale Lugano, Ticino, Switzerland

**Keywords:** stress shielding, bone resorption, RTSA, TSA, reverse total shoulder arthroplasty (RTSA), total shoulder arthroplasty (TSA), cortical thinning, greater tuberosity resorption, calcar resorption

## Abstract

**Background:**

- Stress shielding of the proximal humerus represents an increasing challenge for the implantation of total shoulder arthroplasty (TSA). The literature on this topic is heterogeneous, and many studies analysing its clinical impact are underpowered, justifying a systematic review of current evidence.

**Materials and methods:**

- On 1 September 2024, the Cochrane Library, PubMed, and Web of Science were searched for studies on stress shielding in shoulder arthroplasty. All studies meeting inclusion criteria were included in the qualitative review. Clinical significance was assessed through a meta-analysis of studies comparing patients with and without stress shielding using Constant–Murley score (CS) and American Shoulder and Elbow Surgeons (ASES) score.

**Results:**

- Of 299 identified studies, 58 studies were included in the qualitative review and 13 studies were included in the meta-analysis. Key risk factors consistently associated with stress shielding were press-fit or long stems and high stem-to-humerus filling ratios (FRs), with threshold values of <0.7 or <0.8. Other potential risk factors include older age, poor bone quality, fracture arthroplasty, and stem length. The meta-analysis showed a statistically significant 5.6-point improvement in Constant scores for the non-stress shielding group at two years, although this fell below the minimal clinically important difference threshold and did not affect revision rates at mid-term follow-up.

**Conclusion:**

- Stress shielding is a frequent radiographic finding in TSA and RTSA. Although statistically significant, its clinical impact appears limited, and revision rates remain unaffected in the mid-term. Avoiding high FRs and careful implant selection may help reduce its occurrence and preserve bone stock.

## Introduction

The use of shoulder arthroplasty has expanded rapidly, from fracture arthroplasty and rotator cuff tear arthropathy to primary osteoarthritis (1, 2). The high prevalence of shoulder arthroplasty in the global population has raised new concerns about possible complications that may influence prognosis and lead to more complex revision surgery (3). Among these, the observation of bone resorption around the stem, attributed to the stress shielding effect, has stimulated a strong interest, particularly in relation to an increase in uncemented, press-fit implants (1), which have been shown to further increase bone resorption (4, 5, 6, 7).

In addition to the use of modern press-fit stems, several risk factors for stress shielding have recently been identified, including a high stem-to-humerus filling ratio, longer stems, and fracture arthroplasty (4, 5, 7, 8, 9, 10). Emerging evidence indicates that short-stem and stemless designs may reduce stress shielding by preserving proximal bone stock and more closely mimicking native biomechanics (11). Furthermore, recent studies have explored the use of autologous bone grafts, with the aim of reducing the stem-to-humerus filling ratios and thereby mitigating stress shielding in primary shoulder arthroplasty (12, 13). However, the clinical significance of these interventions remains poorly understood, with limited long-term follow-up data available (12, 13). Interpretation of existing evidence is further complicated by inconsistencies in classification systems (grading and zoning) (8) and imaging modalities (14) (radiographs or computed tomography) used to assess stress shielding, leading to challenges in comparing outcomes across studies (8).

The primary aim of this systematic review is to identify factors influencing the development of stress shielding at the proximal humerus in shoulder arthroplasty. Secondarily, we will evaluate assessment modalities and classification systems used to describe stress shielding. Finally, a meta-analysis will be conducted to quantify the clinical impact of stress shielding on implant performance and its association with complications in short- and mid-term follow-up.

## Methods

### Search strategy and selection criteria

This study was registered on PROSPERO (CRD42024585085) and conducted in accordance with PRISMA guidelines. A systematic database search was performed on 1 September 2024 in PubMed/MEDLINE, the Cochrane Library, EMBASE, and Web of Science with the following search string: (“bone resorption” OR “stress shielding” OR “stressshielding”) AND (“reverse total shoulder arthroplasty” OR “RTSA” OR “total shoulder arthroplasty” OR “shoulder prosthesis” OR “shoulder arthroplasty”). Reference lists in review articles identified during this search, as well as those of the final included articles, were screened to identify additional potentially eligible studies. The inclusion criteria were original clinical or preclinical biomechanical or finite element studies, studies reporting data on stress shielding in proximal humerus following shoulder arthroplasty for any underlying pathology, and articles written in English; no data restrictions were applied. The exclusion criteria were narrative or systematic review, preclinical studies (except biomechanical or finite element analyses), or studies on shoulder arthroplasty that did not report on stress shielding.

### Data analysis and risk of bias assessment

After removing duplicates, two reviewers (MK and DP) independently screened articles in two stages: first by title, abstract, and keywords and then full-text review. Any conflicts were resolved through discussion or by consultation with a third independent reviewer (ML). Data extraction was performed by one reviewer (MK) using an extraction table, with all data points independently verified by a second reviewer (DP). The methodological quality of the included studies was assessed using the Newcastle–Ottawa scale (NOS) (15).

For inclusion in the meta-analysis, studies were required to compare clinical outcomes or complications in patients with and without stress shielding, with an appropriate control group and a minimum follow-up of two years.

### Data extraction and outcome measures

The following demographic and background data were extracted from the included studies: level of evidence, study design, inclusion and exclusion criteria, the number of patients screened, included, and lost to follow-up, mean follow-up duration, gender distribution, age, underlying condition leading to shoulder arthroplasty, stem fixation method, stem length, and prosthesis type (anatomic or reverse).

The following clinical and radiological outcomes were recorded: Constant–Murley score (CS), American Shoulder and Elbow Surgeons (ASES) score, Disabilities of the Arm, Shoulder and Hand (DASH) score and the prevalence of patients with stress shielding and/or high-grade stress shielding. Since various grading systems for stress shielding exist (low grade vs high grade, partial thickness vs full thickness and low adaptive changes vs high adaptive changes), all studies were reclassified using a dichotomous classification: low-grade stress shielding (including partial-thickness, low adaptive changes) vs high-grade stress shielding (including full-thickness, high adaptive changes) to minimise heterogeneity and improve result interpretation. Where possible, existing classifications and measurement approaches were aligned with the framework proposed by Denard *et al.* (8) and major differences were explicitly highlighted. Where standard deviations were not reported, they were estimated using the method by Wan *et al.* (16).

To synthesise the retrieved data, both qualitative and quantitative syntheses were conducted. The qualitative synthesis summarises the evidence regarding the methods used to evaluate stress shielding (including stress shielding grades and zones, implant description, and radiological measures), the identified risk factors for stress shielding (both patient- and implant-related), the reported risk of developing stress shielding over time, and its reported clinical impact. To better quantify the clinical relevance of stress shielding, a meta-analysis was then conducted, comparing patients with and without stress shielding after shoulder arthroplasty and clinical outcome using CS (primary outcome) and ASES and DASH. The postoperative follow-up time points were set as 2 years (2–3 years) and 5 years (4–7 years) after surgery. Sub-analyses were performed for the dichotomous classification of recorded stress shielding (low-grade stress shielding vs high-grade stress shielding).

### Statistical analysis

To quantify the effect of stress shielding and high-grade stress shielding, z-tests for the pooled mean difference (continuous variables) and the pooled risk ratio (dichotomous variables) were used. The heterogeneity of the studies was tested using Cochran’s Q test and the *I*^2^ index. An *I*^2^ > 25% was considered an indicator of significant heterogeneity. For *I*^2^ < 25%, a fixed-effects model was used; otherwise, a random-effects model was applied. A *P*-value <0.05 was considered statistically significant. All statistical analyses were performed using R (R: A Language and Environment for Statistical Computing, R Foundation for Statistical Computing, Vienna, Austria. URL: https://www.R-project.org/) with the *meta* package.

## Results

**Figure 1:**
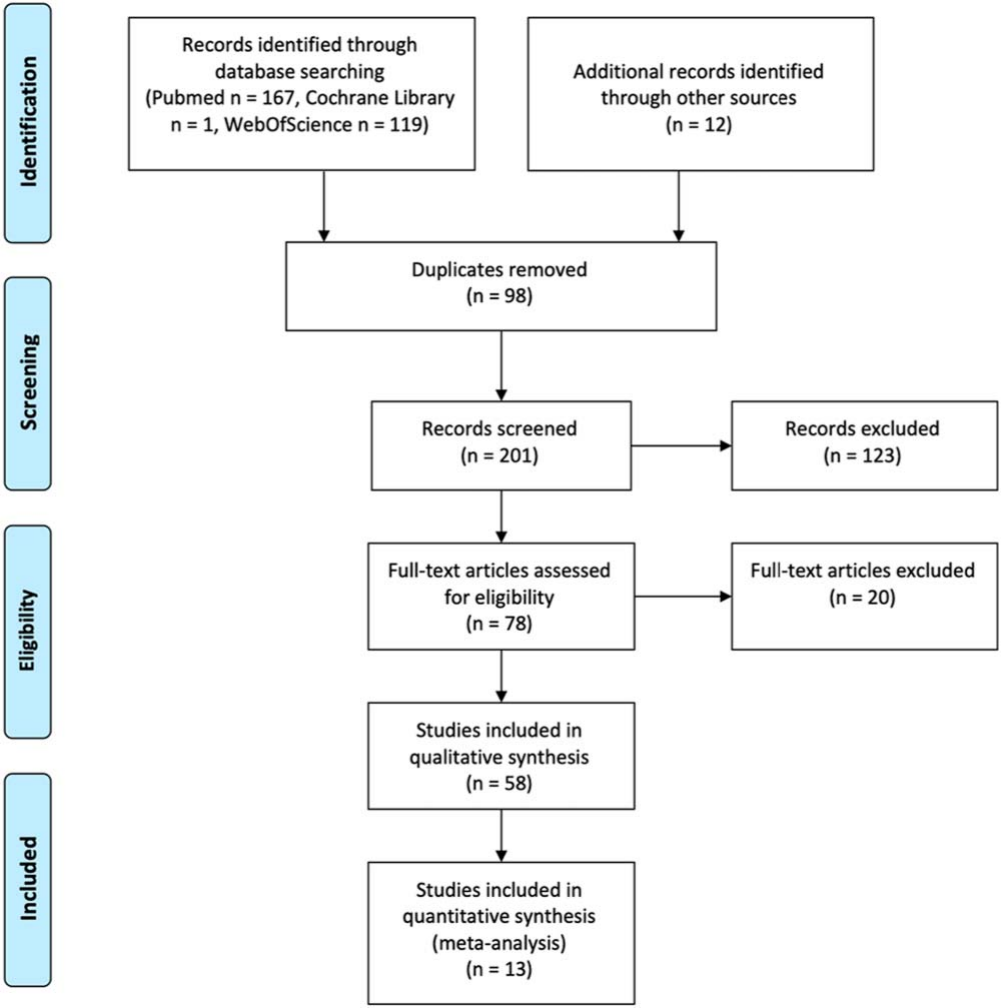
PRISMA flow diagram of the systematic search process, including the study inclusion and exclusion criteria.

The search identified 287 articles, with an additional 12 articles identified through references screening. After removing 98 duplicates, 201 articles were screened, of which 58 studies were included in the qualitative review and 13 studies were included in the quantitative meta-analysis (7, 17, 18, 19, 20, 21, 22, 23, 24, 25, 26, 27, 28) (Fig. 1). In two of the included studies, populations were divided into subgroups due to the availability of standard deviations only at subgroup level. In particular, Mazaleyrat *et al.* (21) separated cemented and uncemented cohorts and García-Fernández *et al.* (23) differentiated between implant types. Study population sizes and demographics characteristics are summarised in Table 1.

**Table 1:** List of studies included in the meta-analysis, including important basic information and risk of bias assessment.

Study	Year	Fixation	Stem length	Prosthesis type	NOS	Pts, *n*	Age	MFUP, years	Female %	SSH %	hgSSH %
Aibinder *et al.* (26)	2023	Uncem	Stemless	Anatomic	8	152	61	2	47	40	7
Gunst *et al.* (24)	2024	Uncem	Standard	Reverse	8	70	78.2	3.25	78	79	NA
Kramer *et al.* (7)	2023	Uncem	Mixed	Reverse	7	50	70.6	2	64	40	NA
Schnetzke *et al.* (19)	2016	Uncem	Short	Anatomic	7	52	71.6	2	60	52	NA
Nourissat *et al.* (18)	2022	Uncem	Standard	Reverse	6	19	74.6	6	78	26	NA
Spormann *et al.* (24)	2014	Uncem	Standard	Anatomic	6	71	67.8	5	63	17	NA
Schnetzke *et al.* (20)	2018	Uncem	Short	Anatomic	7	67	71	5.4	64	91	37
Mazaleyrat *et al.* (21)											
Uncem	2021	Mixed	Standard	Reverse	9	56	83.5	9.6	76	32.1	NA
Cem	2021	Mixed	Standard	Reverse	9	56	83.9	9.4	78	66	NA
Cole *et al.* (28)	2020	Uncem	Standard	Anatomic	7	47	65.3	6.6	47	NA	31.9
Raiss *et al.* (22)	2014	Mixed	Standard	Anatomic	6	395	NA	8	NA	43	23.3
Heuberer *et al.* (25)	2018	Uncem	Stemless	Anatomic	7	53	67.7	2.31	66	47	NA
García-Fernández *et al.* (23)											
Delta Xtend	2024	Uncem	Standard	Reverse	6	33	75.4	6.75	82	51.6	NA
Lima	2024	Uncem	Standard	Reverse	6	35	75.6	6.61	86	60	NA

Pts, patients; NOS, Newcastle–Ottawa scale; and MFUP, mean follow-up.

### Classification of stress shielding and related factors

Nagels *et al.* were among the first to raise awareness of stress shielding as an implant-associated complication in shoulder arthroplasty (17), leading to various procedural and technical techniques and theories published in the subsequent literature to address this issue. Later, Denard *et al.* emphasised the need for a standardised classification system (8).

#### Stress shielding grades

**Figure 2:**
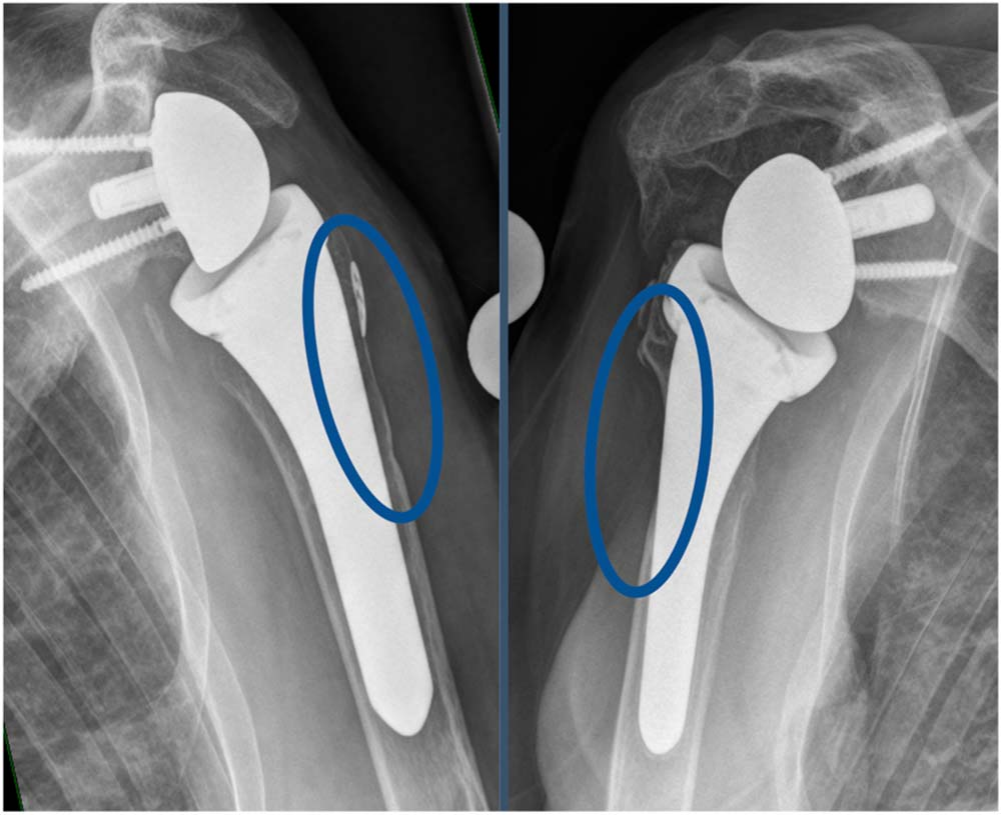
Representative images illustrating the two grades of stress shielding: low grade (left; partial cortical thinning) and high grade (right; full cortical thinning).

Multiple poorly delineated grading systems have been reported in the literature, attempting to categorise stress shielding into either two groups (no cortex thinning vs evidence of cortex thinning (4, 18, 19, 23, 24) or low-grade vs high-grade changes (28)) or three groups (no vs mild vs severe (20, 26) or no vs partial cortical thickness vs full cortical thickness (7, 22, 25)). We recommend a three-category system: ‘no stress shielding’, ‘low-grade stress shielding’, and ‘high-grade stress shielding’, regardless of anatomical location. Minimal differences in density in cancellous bone are not relevant for clinical use or future research and should therefore be classified as ‘no stress shielding’. However, as soon as the cortex appears thinned on conventional radiographs, this should be classified as low-grade stress shielding, regardless of whether it occurs in the metaphyseal or diaphyseal region. Higher-grade bone resorption should only be classified as high-grade stress shielding if the prosthesis stem is completely exposed at least at one point on the conventional radiograph. The analysis should be performed on at least one anteroposterior radiograph or, ideally, additionally on an axillary view. Figure 2 shows an example of low-grade stress shielding and high-grade stress shielding at the typical position in the lateral proximal area for better understanding (Fig. 2).

#### Stress shielding zones

**Figure 3:**
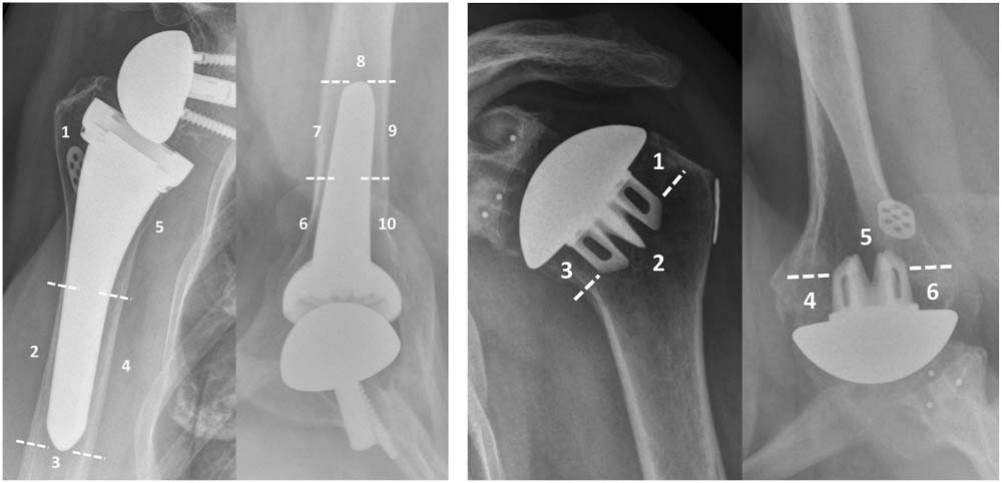
(A and B) Anteroposterior and axillary views of a stemmed reverse shoulder prosthesis (A) showing division into 10 zones (5 + 5). Anteroposterior and axillary views of a stemless anatomic total shoulder prosthesis (B) showing division into 6 zones (3 + 3).

Some studies broadly define zones as medial/lateral or specify regions like the greater tuberosity and calcar (24). Others define zones more precisely, with 7 or 8 zones for standard stems (18, 23, 27, 29, 30) and 5 zones for short stems (19, 29). Denard *et al.* proposed a zoning system using standardised anteroposterior and axillary radiographs, with 5 zones (or 3 zones for stemless implants) on each view (Fig. 3A and B), a system widely adopted by other research groups (7, 8, 28). Figure 3A shows the zones for a standard and short stem on both the anteroposterior and axillary radiograph. Figure 3B shows the zones for stemless implants, the lateral (1 and 2) and medial (4 and 5) zones merge on the anteroposterior radiograph, and the anterior (6 and 7) and posterior (9 and 10) zones merge on the axillary radiograph. The zones at the tip of the prosthesis remain unchanged.

#### Stem-to-humerus filling ratio

**Figure 4:**
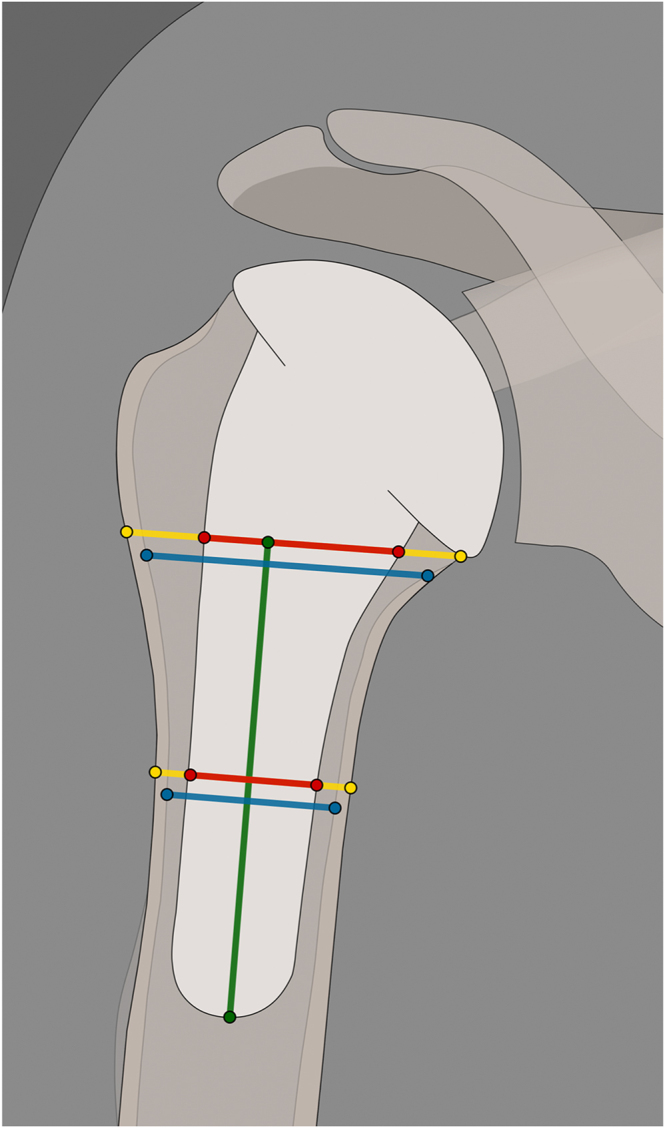
The filling ratio is measured at the level of the medial calcar and midway between the proximal measurement and the prosthetic tip. Stem diameter (red) is divided by either the inner cortical diameter (blue) or outer cortical diameter depending on the measurement method.

Most studies report the metaphyseal filling ratio (mFR) or distal filling ratio (dFR), measured at the metaphysis or midpoint between the medial calcar and the stem tip (7, 8, 14, 18, 19, 20, 24). There is debate about using the inner or outer cortical diameter (8, 31); however, inner cortical measurements have been shown to more accurately identify clinically relevant stress shielding (7). Figure 4 shows both measurements. Raiss *et al.* demonstrated that radiograph-derived measurements underestimate the dFR by an average of ∼3% compared to CT-based methods (14).

### Qualitative review

Risk factors for the occurrence of stress shielding can be broadly divided into patient-related and implant-/procedure-related risk factors. In the following section, all risk factors investigated are addressed and the available literature is summarised.

#### Mechanical risk factors from finite element analyses

Five finite element studies explored mechanical risk factors for stress shielding. Three studies linked increased stem width or length to stress shielding (32, 33, 34). Quental *et al.* found that reduced muscle activation, poor bone quality, and uncemented stems worsened stress shielding, although it was not a major cause of implant failure (35). Tavakoli demonstrated that inferomedial head positioning increases medial cortical stress at the expense of trabecular loading, whereas superolateral positioning shifted stress laterally (36). All finite element studies recommend clinical validation of their findings.

#### Patient-related risk factors

##### Age

Six studies investigated age as a variable. Kramer *et al.* (7) found no significant age difference in patients with versus without stress shielding in uncemented RTSA, a result confirmed by others (37, 38). Spormann *et al.* found a significant mean age difference (71 vs 67 years) in patients with stress shieling and uncemented anatomic implants, although they did not perform further multivariate analysis (27). Peduzzi *et al.* and Kleim *et al.* reported associations in short-stem prostheses; however, age was not significant in multivariate analysis (39, 40).

##### Sex

Female sex was associated with higher stress shielding in bivariate analyses (40, 41), but this was not confirmed in multivariate models. Several studies found no correlation between sex and stress shielding (27, 37, 38).

##### Bone quality

Kramer *et al.* reported no difference in deltoid tuberosity indices (7); however, Abduh *et al.* (42) associated a higher Tingart cortical index (TCI > 2.9 mm) with lower stress shielding, and Peduzzi *et al.* found lower TCI values correlated with stress shielding in their bivariate analysis (39).

#### Implant and procedural risk factors

##### Fracture RTSA

There are no direct comparisons between fracture and non-fracture RTSA with the same prosthesis model with regard to stress shielding, and the frequency of occurrence in fractures cases varies in the literature from 24 to 100% due to the different measurement methods and prosthesis designs (6, 37, 43, 44, 45). However, when a cohort of fracture RTSA of Kramer *et al.* is compared with a cohort of non-fracture RTSA of the same uncemented standard stem, there is a clear higher frequency of low-grade stress shielding (100 vs 52%) and especially high-grade stress shielding (65 vs 7%) in fracture arthroplasty (6, 7). Furthermore, regarding the incidence of stress shielding in fracture RTSA, Porcellini *et al.* reported lower stress shielding values with anatomically healed greater tuberosity (45).

##### Cemented vs uncemented stems

In a computational bone remodelling model, Quental *et al.* mathematically calculated the force transfer from the humerus to the stem after implantation of cemented and uncemented stems. These analyses showed that force transmission was higher at the bone–implant interface than at the bone–cement interface, which explains why cemented stems showed less bone resorption in their subsequent bone remodelling analysis (35). These observations have been confirmed in clinical cases, with approximately double the rates of stress shielding in the uncemented cohorts compared to cemented implants (4, 9, 21). Overall, however, the frequency reported in the included studies varies from 0 to 32% for cemented stems and from 14 to 100% for uncemented stems, depending on the indication, implant type, or measurement method (5, 6, 11, 43, 46, 47, 48, 49). In a multivariate regression analysis, Yokoya *et al.* identified an uncemented stem as the single most important predictor of the presence of stress shielding, with a partial regression coefficient of 6.04 (*P* = 0.01) and an odds ratio of 422 (11).

##### Stem length

Most studies showed that the risk of stress shielding increases with longer stems with a reported frequency of 0–41% (38, 47, 50, 51, 52, 53) and bone resorption mainly occurring around the medial calcar in stemless implants (47). There are mainly comparative studies between short and standard stems. Erickson *et al.* analysed a cohort with both RTSA and TSA, finding a 2.2% incidence of stress shielding around the greater tuberosity and 2.9% near the medial calcar for short stems and 5.8 and 12.9%, respectively, for standard stems one year after implantation (54). After at least 2 years of follow-up, however, frequencies of 21–50% are reported for short stems and 52–77% for standard stems (7, 55, 56). The multivariate regression for independent risk factors of Yokoya *et al.* showed the stem length to be a significant influencing factor (*P* = 0.005) but with a rather small effect (regression coefficient of 0.12 and an odds ratio of 0.88) (11).

##### Stem design

Regarding implant coating, Inoue *et al.* found in their regression analysis that the on-growth-type coating in anatomic shoulder replacements was a significant risk factor for stress shielding. However, a subsequent study of the same research group did not confirm these results in a cohort of reversed shoulder replacements (30, 41). Giordano *et al.* found an influence of stem design on stress shielding with higher rates occurring in a 145° inlay and curved, collarless, circular stems, compared to a 155° inlay and straight stems. The first implant design mentioned showed stress shielding medially in 35% and laterally in 18%, while the second implant design showed stress shielding medially in 17% and laterally in 7% (57). Stress shielding was significantly more often in a collarless and curved stem design compared to a straight and collared stem design for the lateral metaphysis (79 vs 20%; *P* = 0.001), the lateral diaphysis (53 vs 11%; *P* = 0.002), the medial metaphysis (95 vs 63%; *P* = 0.011), and the medial diaphysis (50 vs 14%; *P* = 0.009) (58).

##### Reverse vs anatomic total shoulder replacement

There are limited data available comparing the occurrence of stress shielding with implant type (RSA, hemiarthroplasty, or TSA). Most studies found no difference between RTSA and TSA in multivariate analysis (39, 40, 42). However, one study reported higher stress shielding rates in hemiarthroplasty compared to TSA (30).

##### Filling ratios

Various studies have shown that stem-to-humerus filling ratios are among the most important risk factors for stress shielding (7, 8, 10, 12, 19, 31). Initially reported by Nagels *et al.* (17) for shoulder hemiarthroplasty, this finding has since been reproduced by multiple subsequent studies, including when measured at different levels (metaphyseal and distal measurement), as well as in TSA and RTSA and for fractures (7, 10, 11, 12, 13, 19, 20, 27, 30, 31, 37, 39, 40, 59, 60, 61). A correlation between the three-dimensionally measured filling ratios and the occurrence of stress shielding has also been demonstrated (59). Raiss *et al.* analysed a TSA cohort, comparing cases with a dFR greater than and less than 0.7, and found a fourfold lower occurrence of stress shielding in the latter group. Similarly, in a RTSA cohort, sevenfold less stress shielding with a dFR lower than 0.8 was found (7, 31). Kramer *et al.* calculated an optimised combined cut-off for metaphyseal and distal filling ratios of 0.7 with recursive partitioning. When both thresholds were applied, stress shielding was reduced tenfold (7). An even lower limit of 0.55 was postulated by Kleim *et al.* for the average of mFR and dFR, which was based on a modified measurement method (40).

Due to this evidence, implantation techniques have been modified to actively minimise filling ratios during stem implantation. Kim *et al.* achieved this by using autologous bone grafting from the humeral head (13), and Montemaggi *et al.* described a successful humeral matchstick autograft augmentation technique (12). According to their findings, stress shielding rates were significantly reduced, without compromising stem stability. Interestingly, both the clinical scores and the range of motion in the low filling ratio group were significantly worse than in the high filling ratio group in the cohort of Kim *et al.*, making the use of bone graft a subject of ongoing debate (13).

##### Other risk factors

In addition to the previously reported patient and implant characteristics, other risk factors for stress shielding were investigated by individual papers. Aibinder *et al.* showed a correlation between subscapularis osteotomy and reduced stress shielding compared to peel-off and tenotomy in cementless stemless TSA (26), and Endell *et al.* found no association between stress shielding and sporting activities after TSA (62). Spormann *et al.* showed that stress shielding occurs more frequently in post-traumatic than in primary osteoarthritis (27), and Peduzzi *et al.* demonstrated in a multivariate regression analysis that a varus deviation of the stem is an independent risk factor for stress shielding on the medial side, whereas valgus deviation is a risk factor on the lateral side (39). A finite element analysis by Mueri *et al.* has also shown that tilting the stem into a varus position, even if only by a few degrees, leads to a significant increase in stress shielding (63).

##### Progression of stress shielding over time

Spormann *et al.* reported a 5-year long-term follow-up of 132 patients treated with press-fit TSA. Of those, 22 demonstrated full-thickness stress shielding after 12 months, with three more cases occurring after 24 months. No further cases were observed thereafter. Within their case series, the spatial extent of stress shielding increased from 10 mm at 6 months to 20 mm at 3 years (27). Similarly, other studies of short stems and trauma RTSAs found no increase in stress shielding between 2 and 5 years (20) and a significant progression of stress shielding after 2 years has not yet been shown in the current literature.

##### Clinical outcomes and revisions due to stress shielding

The majority of the included studies showed a statistically non-significant trend toward better function in association without stress shielding (6, 7, 17, 19, 20, 21, 23, 25, 26, 27, 28). Garcia-Fernandez *et al.* (23) found a statistically non-significant improvement of the Constant score in patients with stress shielding, whereas others showed significantly better CS (22, 24) or ASES (18) scores in the non-stress shielding group. The majority of significant findings did not exceed the minimal clinically important difference (MCID). Of the included studies, none showed a higher revision rate in the stress shielding groups.

### Quantitative review and meta-analysis

Three meta-analyses were performed according to existing data to assess the functional outcome scores in relation to the prevalence of stress shielding in total (low grade and high grade vs no stress shielding) or high-grade stress shielding vs no high-grade stress shielding, as follows:

- Constant score comparison between patients with vs without stress shielding after 2 and 5 years.
- ASES score comparison between patients with vs without stress shielding after 2 and 5 years.
- ASES score comparison between patients with vs without high-grade stress shielding after 2 and 5 years.

Subgroup analyses were performed whenever possible based on the available data. Comparison between low-grade and high-grade stress shielding with regard to the Constant score was not possible due to a lack of data in the literature. Furthermore, there were insufficient data to allow a comparison of the DASH scores.

**Figure 5:**
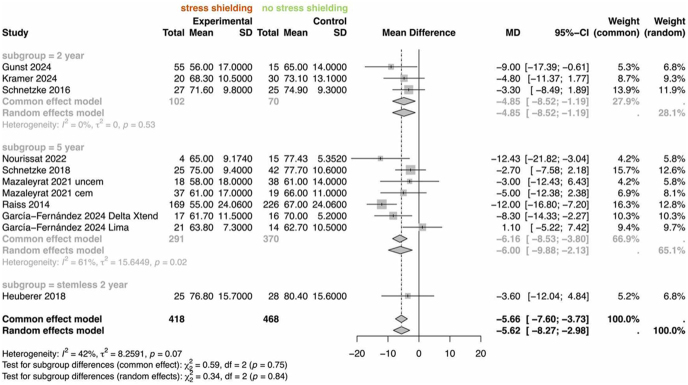
Forest plot of the meta-analysis for Constant–Murley score outcomes in patients with and without stress shielding. Subgroups include short or standard stems at 5 years and stemless implants at 2 years.

The meta-analysis on Constant score shows an overall statistically significant mean difference of 5.6 points (95% CI: 8.3–3.0, *P* < 0.001), with worse outcomes in the stress shielding group. The results are statistically significant for stemmed implants at both the 2-year (mean: 4.9, 95% CI: 8.5–1.2, *P* < 0.001) and the 5-year (mean: 6.0, 95% CI: 9.9–2.1, *P* < 0.001) follow-ups, respectively. There was no significant association in the stemless subgroup; however, only one study with a 2-year follow-up period was included (Fig. 5).

**Figure 6:**
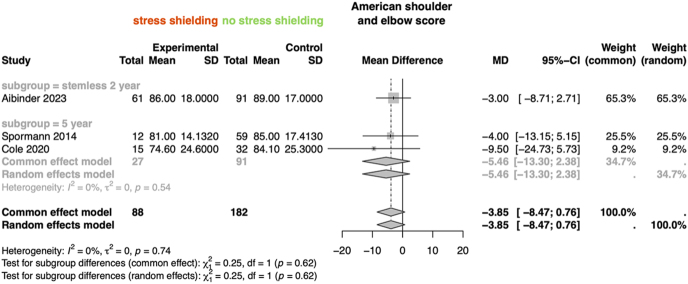
Forest plot of the meta-analysis for ASES outcomes in patients with and without stress shielding. Subgroups include short or standard stems at 5 years and stemless implants at 2 years.

**Figure 7:**
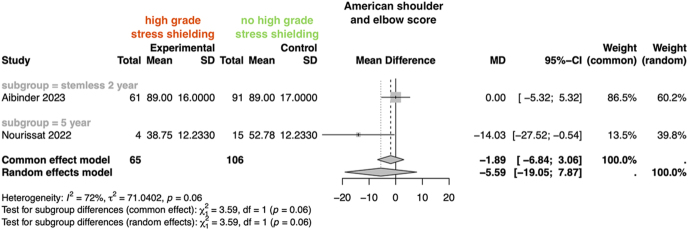
Forest plot of the meta-analysis for ASES outcomes in patients with and without high-grade stress shielding. Subgroups include stemless implants at 2 years and standard stems implants at 5 years.

The result of the analysis on ASES score found no statistically significant difference between the non-stress shielding and the stress shielding groups or the high-grade vs no high-grade stress shielding group (Figs 6 and 7).

## Discussion

Stress shielding following shoulder arthroplasty is a frequently observed radiographic finding, yet its clinical significance remains unclear. This systematic review synthesised current evidence on prevalence, risk factors, and potential clinical impact. Findings indicate that stress shielding predominantly develops within the first two years postoperatively (20, 27), particularly in association with uncemented, longer stems and higher stem-to-humerus filling ratios, both metaphyseal and distal (7, 8, 11, 14, 18, 19, 20, 24).

Our meta-analysis revealed a statistically significant association between stress shielding and inferior Constant–Murley scores at 2- and 5 year follow-up intervals. However, the observed difference fell below the MCID threshold (64), calling into question its clinical relevance. Furthermore, no increase in revision rates was identified within the first five postoperative years. This suggests that although the presence of stress shielding may influence functional outcomes to a limited extent, it may not translate into early implant failure. Long-term data from large-scale registry-based studies are needed to clarify potential consequences, including periprosthetic fracture risk and revision burden.

Stress shielding is believed to be mechanistically driven, particularly during the early ‘run-in’ phase of implant integration (typically up to 2 years). Changes in the load distribution due to different material properties of bone and prosthesis stem lead to stress shielding in the proximal bone. As the force is transferred through the stem, this results in localised bone resorption due to reduced mechanical stimulation, as described by Wolff’s law (8, 34, 35).

Risk factors can be broadly categorised into ‘implant-associated’ and ‘patient-related’ domains. Implant-related factors include fracture reverse total shoulder arthroplasty, the use of uncemented and longer stems, a higher distal or metaphyseal filling ratio, and specific stem designs (e.g. collarless, curved, circular with on-growth-type coating). Patient-related factors, such as older age, female sex and compromised bone quality, have also been implicated.

However, multivariate regression analyses failed to confirm the statistical significance of patient-related variables. This likely reflects clinical practice wherein surgeons may compensate for lower bone quality (often found in elderly or female patients) by selecting larger stems to ensure implant stability, inadvertently increasing the filling ratio (7). In addition, systemic factors, such as hormonal changes and age-related bone turnover, may confound these associations (65). Although causal relationships remain speculative, these variables may help identify higher-risk patients, for whom mitigating strategies should be prioritised.

Among modifiable risk factors, stem implantation technique (e.g. cemented vs press-fit) (5, 6, 11, 43, 46, 47, 48, 49), stem length (11), and filling ratios (7, 8, 31) are of particular relevance. Finite element analyses support the hypothesis that cemented fixation achieves a more uniform stress distribution, potentially reducing bone remodelling around the implant (35). Longer stems not only tend to increase filling ratios but also independently contribute to stress shielding (7, 11). Both metaphyseal and distal filling ratios above 0.7–0.8 have been consistently associated with increased stress shielding, regardless of the measurement method (7, 8, 14).

Current evidence suggests that reducing the filling ratio, either directly or through the use of bone grafts, or adopting cemented fixation are the only known surgical strategies to mitigate stress shielding (11, 12, 13). However, the impact of these approaches on long-term clinical outcomes and implant stability remains poorly understood (12, 13). These measures may be most appropriate in high-risk patients or in clinical scenarios necessitating high primary stability (e.g. fracture arthroplasty) (6, 66). Further efforts should also consider optimisation of stem designs, such as stemless or metaphyseal-anchoring implants, and refined surgical techniques to minimise stress shielding. In conclusion, these findings underscore the need for targeted strategies aimed at at-risk patient populations, as well as further prospective studies to assess the long-term implications of stress shielding on implant survivorship and clinical outcomes.

### Limitations

This systematic review and meta-analysis has several notable limitations. First, the majority of included studies were retrospective cohort studies (level III evidence), which introduces an inherent risk of bias and limits the strength of causal inferences. To mitigate this, we applied the Newcastle–Ottawa scale (NOS) for quality assessment, including only studies that met a minimum threshold for methodological rigour, and we interpreted findings with appropriate caution. Second, there was considerable variation in the terminology, grading systems, and imaging modalities (e.g. radiographs vs CT) used to evaluate stress shielding across studies. To address this, we standardised the classification of stress shielding using a dichotomous grading system (low grade vs high grade), based on existing frameworks (e.g. Denard *et al.* (8)), to improve data synthesis and reduce variability in interpretation. We acknowledge that other measurement methods for stress shielding exist, some of which allow for more quantitative assessment. However, using these alternative approaches would not allow for a meaningful comparison with the existing literature, given the heterogeneity of methodologies and reporting standards. The phenomenon of stress shielding remains difficult to characterise and quantify reliably. Reported incidence rates in the literature range widely, reflecting methodological differences and the challenges in distinguishing between no, low-grade, and high-grade stress shielding. Moreover, diagnosis may be confounded in osteoporotic or post-traumatic bone, where differentiation is particularly challenging. Therefore, current evidence regarding the true frequency and clinical relevance of stress shielding should be interpreted with caution, and our findings must be viewed within this context of uncertainty. Third, due to the lack of long-term data, the study could not adequately evaluate the impact of stress shielding on late complications, such as periprosthetic fractures or revision surgery beyond five years. We highlighted this gap and called for future registry-based studies with extended follow-up to more accurately assess the clinical consequences of stress shielding. Finally, the meta-analyses were constrained by the limited availability of standardised outcome data (particularly for ASES and DASH scores), which reduced statistical power and the ability to draw robust conclusions about clinical outcomes. Therefore, and to maximise analytical robustness, we focused the meta-analyses on Constant–Murley scores, which were more consistently reported, and transparently reported the limitations in the available outcome data.

## Conclusion

This review highlights that stress shielding is a common radiographic finding after shoulder arthroplasty, primarily driven by mechanical factors such as uncemented fixation, longer stems, and high filling ratios. Although associated with lower functional scores, the clinical relevance appears limited, and no increase in early revision rates has been observed. Surgical technique and implant choice remain key modifiable factors. Longer-term studies are needed to clarify its impact on implant longevity.

## ICMJE Statement of Interest

The authors declare that there is no conflict of interest that could be perceived as prejudicing the impartiality of the work reported.

## Funding Statement

Each author certifies that there are no funding or commercial associations (consultancies, stock ownership, equity interest, patent/licensing arrangements, etc.) that might pose a conflict of interest in connection with the submitted article related to the author or any immediate family members.

## Author contribution statement

All authors contributed to the study conception and design. Statistical analyses were performed by MK and DP. The first draft of the manuscript was written by MK and DP and revised several times in cooperation with ML and BJ. All authors read and approved the final manuscript and believe that the manuscript represents honest work.

## References

[1] Australian Orthopaedic Association National Joint Replacement Registry (AOANJRR). In Hip, knee and shoulder arthroplasty: 2022 Annual Report. Adelaide, Australia: AOA, 2022. (https://aoanjrr.sahmri.com/annual-reports-2022)

[2] Villatte G, Erivan R, Barth J, et al. Progression and projection for shoulder surgery in France, 2012–2070: epidemiologic study with trend and projection analysis. Orthopaedics Traumatol Surg Res 2020 106 1067–1077. (10.1016/j.otsr.2020.04.019) 32863170

[3] Farley KX, Wilson JM, Kumar A, et al. Prevalence of shoulder arthroplasty in the United States and the increasing burden of revision shoulder arthroplasty. JBJS Open Access 2021 6 e20.00156. (10.2106/jbjs.oa.20.00156) PMC8280071 34278185

[4] Mazaleyrat M, Favard L, Boileau P, et al. Humeral osteolysis after reverse shoulder arthroplasty using cemented or cementless stems comparative retrospective study with a mean follow-up of 9 years. Orthopaedics Traumatol Surg Res 2021 107 102916. (10.1016/j.otsr.2021.102916) 33812096

[5] Brolin TJ, Cox RM, Horneff JG, et al. Humeral-sided radiographic changes following reverse total shoulder arthroplasty. Arch Bone Joint Surg 2020 8 50–57. (10.22038/abjs.2019.36065.1951) 32090146 PMC7007723

[6] Kramer M, Olach M, Zdravkovic V, et al. Cemented vs uncemented reverse total shoulder arthroplasty for the primary treatment of proximal humerus fractures in the elderly – a retrospective case–control study. BMC Musculoskelet Disord 2022 23 1043. (10.1186/s12891-022-05994-3) 36457072 PMC9714093

[7] Kramer M, Olach M, Zdravkovic V, et al. The effects of length and width of the stem on proximal humerus stress shielding in uncemented primary reverse total shoulder arthroplasty. Arch Orthop Trauma Surg 2024 144 663–672. (10.1007/s00402-023-05129-w) 38010377 PMC10822783

[8] Denard PJ, Raiss P, Gobezie R, et al. Stress shielding of the humerus in press-fit anatomic shoulder arthroplasty: review and recommendations for evaluation. J Shoulder Elbow Surg 2018 27 1139–1147. (10.1016/j.jse.2017.12.020) 29422391

[9] Denard PJ, Haidamous G, Gobezie R, et al. Short-term evaluation of humeral stress shielding following reverse shoulder arthroplasty using press-fit fixation compared with cemented fixation. J Shoulder Elbow Surg 2020 29 906–912. (10.1016/j.jse.2019.09.042) 31911215

[10] de Joode SGCJ, Kriechling P, Volp AS, et al. The effect of humeral diaphyseal stem filling ratio on clinical and radiological outcome. Semin Arthroplasty JSES 2024 34 340–347. (10.1053/j.sart.2023.12.004)

[11] Yokoya S, Harada Y, Sumimoto Y, et al. Factors affecting stress shielding and osteolysis after reverse shoulder arthroplasty: a multicenter study in a Japanese population. J Orthop Sci 2024 29 521–528. (10.1016/j.jos.2023.01.003) 36710212

[12] Montemaggi P, Lo EY, Ouseph A, et al. Cementless reverse total shoulder arthroplasty implantation with humeral matchstick autograft augmentation: early radiographic outcomes. J Shoulder Elbow Surg 2024 33 e422–e428. (10.1016/j.jse.2023.11.021) 38218403

[13] Kim SC, Park JH, Bukhary H, et al. Humeral stem with low filling ratio reduces stress shielding in primary reverse shoulder arthroplasty. Int Orthop 2022 46 1341–1349. (10.1007/s00264-022-05383-4) 35353240

[14] Raiss P, Wittmann T, Blakeney W, et al. Validation of the distal filling ratio in uncemented convertible short-stem shoulder arthroplasty. Arch Orthop Trauma Surg 2023 143 1833–1839. (10.1007/s00402-022-04389-2) 35174410

[15] Wells GA, Peterson J, Shea B, et al. The Newcastle-Ottawa Scale (NOS) for assessing the quality if nonrandomized studies in meta-analyses [Internet], 2021. [cited 2024 Sep 12]. (https://www.ohri.ca/programs/clinical_epidemiology/oxford.asp)

[16] Wan X, Wang W, Liu J, et al. Estimating the sample mean and standard deviation from the sample size, median, range and/or interquartile range. BMC Med Res Methodol 2014 14 135. (10.1186/1471-2288-14-135) 25524443 PMC4383202

[17] Nagels J, Stokdijk M & Rozing PM. Stress shielding and bone resorption in shoulder arthroplasty. J Shoulder Elbow Surg 2003 12 35–39. (10.1067/mse.2003.22) 12610484

[18] Nourissat G, Corsia S, Harris HW, et al. Specific design of a press fit humeral stem provides low stress shielding in reverse shoulder arthroplasty at minimum 5 years FU. J Shoulder Elb Arthroplast 2022 6 247154922211125. (10.1177/24715492221112543) PMC9272197 35832511

[19] Schnetzke M, Coda S, Raiss P, et al. Radiologic bone adaptations on a cementless short-stem shoulder prosthesis. J Shoulder Elbow Surg 2016 25 650–657. (10.1016/j.jse.2015.08.044) 26560021

[20] Schnetzke M, Rick S, Raiss P, et al. Mid-term results of anatomical total shoulder arthroplasty for primary osteoarthritis using a short-stemmed cementless humeral component. Bone Joint Lett J 2018 100-B 603–609. (10.1302/0301-620X.100B5.BJJ-2017-1102.R2) 29701085

[21] Mazaleyrat M, Favard L, Garaud P, et al. Press-fit vs cemented humeral stem fixation for reverse shoulder arthroplasty: functional outcomes at a mean follow-up of 9.5 years. J Shoulder Elbow Surg 2021 30 72–79. (10.1016/j.jse.2020.04.052) 32838951

[22] Raiss P, Edwards TB, Deutsch A, et al. Radiographic changes around humeral components in shoulder arthroplasty. J Bone Joint Surg 2014 96 e54. (10.2106/jbjs.m.00378) 24695931

[23] García-Fernández C, Lopiz Y, Garríguez-Pérez D, et al. Do the humeral radiographic changes at 5-year follow-up affect the clinical outcomes of press-fit humeral stems in primary reverse shoulder arthroplasties? Eur J Orthop Surg Traumatol 2024 34 1851–1863. (10.1007/s00590-024-03864-3) 38431896

[24] Gunst S, Cloquell Y, Collotte P, et al. Medium-term clinical and radiographic outcomes of a cementless prosthesis with a 140° neck–shaft angle in reverse total shoulder arthroplasty. J Shoulder Elbow Surg 2024 33 1075–1083. (10.1016/j.jse.2023.08.021) 37777044

[25] Heuberer PR, Brandl G, Pauzenberger L, et al. Radiological changes do not influence clinical mid-term outcome in stemless humeral head replacements with hollow screw fixation: a prospective radiological and clinical evaluation. BMC Musculoskelet Disord 2018 19 28. (10.1186/s12891-018-1945-6) 29357861 PMC5778649

[26] Aibinder WR, Uddin F, Bicknell RT, et al. Stress shielding following stemless anatomic total shoulder arthroplasty. Shoulder Elbow 2023 15 54–60. (10.1177/17585732211058804) PMC9990105 36895609

[27] Spormann C, Durchholz H, Audigé L, et al. Patterns of proximal humeral bone resorption after total shoulder arthroplasty with an uncemented rectangular stem. J Shoulder Elbow Surg 2014 23 1028–1035. (10.1016/j.jse.2014.02.024) 24929745

[28] Cole EW, Moulton SG, Gobezie R, et al. Five-year radiographic evaluation of stress shielding with a press-fit standard length humeral stem. JSES Int 2020 4 109–113. (10.1016/j.jses.2019.11.002) 32195472 PMC7075772

[29] Sperling JW, Cofield RH, O’Driscoll SW, et al. Radiographic assessment of ingrowth total shoulder arthroplasty. J Shoulder Elbow Surg 2000 9 507–513. (10.1067/mse.2000.109384) 11155304

[30] Inoue K, Suenaga N, Oizumi N, et al. Humeral bone resorption after anatomic shoulder arthroplasty using an uncemented stem. J Shoulder Elbow Surg 2017 26 1984–1989. (10.1016/j.jse.2017.04.012) 28688934

[31] Raiss P, Schnetzke M, Wittmann T, et al. Postoperative radiographic findings of an uncemented convertible short stem for anatomic and reverse shoulder arthroplasty. J Shoulder Elbow Surg 2019 28 715–723. (10.1016/j.jse.2018.08.037) 30473242

[32] Langohr GDG, Reeves J, Roche CP, et al. The effect of short-stem humeral component sizing on humeral bone stress. J Shoulder Elbow Surg 2020 29 761–767. (10.1016/j.jse.2019.08.018) 31711829

[33] Razfar N, Reeves JM, Langohr DG, et al. Comparison of proximal humeral bone stresses between stemless, short stem, and standard stem length: a finite element analysis. J Shoulder Elbow Surg 2016 25 1076–1083. (10.1016/j.jse.2015.11.011) 26810016

[34] Synnott S, Langohr GDG, Reeves JM, et al. The effect of humeral implant thickness and canal fill on interface contact and bone stresses in the proximal humerus. JSES Int 2021 5 881–888. (10.1016/j.jseint.2021.05.006) 34505100 PMC8411059

[35] Quental C, Folgado J, Fernandes PR, et al. Bone remodelling analysis of the humerus after a shoulder arthroplasty. Med Eng Phys 2012 34 1132–1138. (10.1016/j.medengphy.2011.12.001) 22221559

[36] Tavakoli A, Spangenberg GW, Reeves JM, et al. The effect of humeral head positioning and incomplete backside contact on bone stresses following total shoulder arthroplasty with a short humeral stem. J Shoulder Elbow Surg 2023 32 1988–1998. (10.1016/j.jse.2023.04.006) 37230287

[37] Giol FG, Parellada CV, Baños FG, et al. Stress shielding: short-term radiological results of the reverse shoulder arthroplasty with an anatomic proximal coated stem in proximal humeral fractures. Arch Orthop Trauma Surg 2024 144 783–790. (10.1007/s00402-023-05169-2) 38141095

[38] Rankin IA, Goffin J, Khan LAK, et al. Stress shielding of the proximal humerus in stemless anatomic total shoulder arthroplasty. Shoulder Elbow 2023 144 783–790. (10.1177/17585732231168391) PMC11512461 39464830

[39] Peduzzi L, Goetzmann T, Wein F, et al. Proximal humeral bony adaptations with a short uncemented stem for shoulder arthroplasty: a quantitative analysis. JSES Open Access 2019 3 278–286. (10.1016/j.jses.2019.09.011) 31891026 PMC6928264

[40] Kleim BD, Garving C & Brunner UH. Cementless curved short stem shoulder prostheses with a proximal porous coating: ingrowth properties at 2–5 years of radiological follow-up with clinical correlation. J Shoulder Elbow Surg 2020 29 2299–2307. (10.1016/j.jse.2020.02.025) 32666922

[41] Inoue K, Suenaga N, Oizumi N, et al. Humeral bone resorption after reverse shoulder arthroplasty using uncemented stem. JSES Int 2020 4 138–143. (10.1016/j.jses.2019.11.007) 32195476 PMC7075776

[42] Abduh W, Berhouet J, Samargandi R, et al. Clinical results and radiological bony adaptations on a cementless short-stem prosthesis – a comparative study between anatomical and reverse total shoulder arthroplasty. Orthopaedics Traumatol Surg Res 2022 108 103262. (10.1016/j.otsr.2022.103262) 35248791

[43] Lopiz Y, García-Fernandez C, Vallejo-Carrasco M, et al. Reverse shoulder arthroplasty for proximal humeral fracture in the elderly. Cemented or uncemented stem? Int Orthop 2022 46 635–644. (10.1007/s00264-021-05284-y) 35034145

[44] Lanzetti RM, Gaj E, Berlinberg EJ, et al. Reverse total shoulder arthroplasty demonstrates better outcomes than angular stable plate in the treatment of three-part and four-part proximal humerus fractures in patients older than 70 years. Clin Orthop Relat Res 2023 481 735–747. (10.1097/corr.0000000000002480) 36383078 PMC10013660

[45] Porcellini G, Montanari M, Giorgini A, et al. Great tuberosity fixation does not affect healing and clinical outcomes in RSA performed in proximal humeral fractures in elderly patients. Musculoskelet Surg 2024 108 107–114. (10.1007/s12306-023-00807-9) 38175393

[46] Melis B, DeFranco M, Lädermann A, et al. An evaluation of the radiological changes around the Grammont reverse geometry shoulder arthroplasty after eight to 12 years. J Bone Joint Surg Br 2011 93 B 1240–1246. (10.1302/0301-620X.93B9.25926) 21911536

[47] Aibinder WR, Bartels D, Sperling JW, et al. Mid-term radiological results of a cementless short humeral component in anatomical and reverse shoulder arthroplasty. Bone Joint Lett J 2019 101-B 610–614. (10.1302/0301-620x.101b5.bjj-2018-1374.r1) 31039055

[48] Padegimas EM, Nicholson TA & Namdari S. Short-term results of a convertible diaphyseal-fit anatomic shoulder arthroplasty system. Arch Bone Joint Surg 2023 11 154–159. (10.22038/ABJS.2022.61438.3025) 37168588 PMC10165205

[49] Nguyen N, Martinez-Catalan N, Songy E, et al. Radiological humeral adaptative changes five years after anatomical total shoulder arthroplasty using a standard- length cementless hydroxyapatite-coated humeral component. Bone Joint Lett J 2021 103-B 958–963. (10.1302/0301-620x.103b5.bjj-2020-1619.r1) 33934651

[50] Bülhoff M, Spranz D, Maier M, et al. Mid-term results with an anatomic stemless shoulder prosthesis in patients with primary osteoarthritis. Acta Orthop Traumatol Turc 2019 53 170–174. (10.1016/j.aott.2019.03.011) 30956025 PMC6599416

[51] Beck S, Patsalis T, Busch A, et al. Long-term radiographic changes in stemless press-fit total shoulder arthroplasty. Z Orthop Unfall 2021 159 274–280. (10.1055/a-1079-6549) 32097955

[52] Magone KM, Leonard A, Savoie FH, et al. Short-term radiographic analysis of a stemless humeral component for anatomic total shoulder arthroplasty. JSES Int 2023 7 285–289. (10.1016/j.jseint.2022.12.020) 36911768 PMC9998871

[53] Vegas A, Cannon D, Lewis S, et al. Impact of humeral stem length on calcar resorption in anatomic total shoulder arthroplasty. J Shoulder Elbow Surg 2024 33 130–138. (10.1016/j.jse.2023.05.033) 37419442

[54] Erickson BJ, Denard PJ, Griffin JW, et al. Initial and 1-Year radiographic comparison of reverse total shoulder arthroplasty with a short versus standard length stem. J Am Acad Orthop Surg 2022 30 E968–E978. (10.5435/jaaos-d-21-01032) 35297792

[55] Denard PJ, Noyes MP, Walker JB, et al. Proximal stress shielding is decreased with a short stem compared with a traditional-length stem in total shoulder arthroplasty. J Shoulder Elbow Surg 2018 27 53–58. (10.1016/j.jse.2017.06.042) 28865965

[56] Uschok S, Magosch P, Moe M, et al. Is the stemless humeral head replacement clinically and radiographically a secure equivalent to standard stem humeral head replacement in the long-term follow-up? A prospective randomized trial. J Shoulder Elbow Surg 2017 26 225–232. (10.1016/j.jse.2016.09.001) 27856267

[57] Giordano MC, Corona K, Morris BJ, et al. Comparative study of 145° onlay curved stem versus 155° inlay straight stem reverse shoulder arthroplasty: clinical and radiographic results with a minimum 2-year follow-up. J Shoulder Elbow Surg 2022 31 2089–2095. (10.1016/j.jse.2022.02.042) 35430369

[58] Denard PJ, Noyes MP, Walker JB, et al. Radiographic changes differ between two different short press-fit humeral stem designs in total shoulder arthroplasty. J Shoulder Elbow Surg 2018 27 217–223. (10.1016/j.jse.2017.08.010) 28965688

[59] Celik H, Chauhan A, Flores-Hernandez C, et al. Three-dimensional volumetric filling ratio predicts stress shielding in short-stem anatomic total shoulder arthroplasty. J Am Acad Orthop Surg 2020 28 1047–1054. (10.5435/jaaos-d-19-00444) 32301819

[60] Larose G, Aibinder WR, Greene AT, et al. Two-year minimum survivorship and radiographic analysis of a pressfit short humeral stem for total shoulder arthroplasty. JSES Int 2024 8 191–196. (10.1016/j.jseint.2023.10.011) 38312300 PMC10837737

[61] Schnetzke M, Preis A, Coda S, et al. Anatomical and reverse shoulder replacement with a convertible, uncemented short-stem shoulder prosthesis: first clinical and radiological results. Arch Orthop Trauma Surg 2017 137 679–684. (10.1007/s00402-017-2673-3) 28337535

[62] Endell D, Audigé L, Grob A, et al. Impact of sports activity on medium-term clinical and radiological outcome after reverse shoulder arthroplasty in cuff deficient arthropathy; an institutional register-based analysis. J Clin Med 2021 10 1–13. (10.3390/jcm10040828) PMC7922026 33670531

[63] Mueri C, Maquer G, Henderson A, et al. Effect of humeral stem sizing and alignment on stress shielding – a virtual cohort study. IEEE Trans Biomed Eng 2025 72 3430–3437. (10.1109/TBME.2025.3568877) 40354196

[64] Louwerens JKG, van den Bekerom MPJ, van Royen BJ, et al. Quantifying the minimal and substantial clinical benefit of the Constant-Murley score and the disabilities of the arm, shoulder and hand score in patients with calcific tendinitis of the rotator cuff. JSES Int 2020 4 606–611. (10.1016/j.jseint.2020.05.001) 32939494 PMC7479032

[65] Drake MT, Clarke BL & Lewiecki EM. The pathophysiology and treatment of osteoporosis. Clin Ther 2015 37 1837–1850. (10.1016/j.clinthera.2015.06.006) 26163201

[66] Schoch B, Aibinder W, Walters J, et al. Outcomes of uncemented versus cemented reverse shoulder arthroplasty for proximal humerus fractures. Orthopaedics 2019 42 E236–E241. (10.3928/01477447-20190125-03) 30707233

